# The efficacy and safety of complementary and alternative medicine for depression: an umbrella review

**DOI:** 10.47626/1516-4446-2024-3705

**Published:** 2024-11-25

**Authors:** Yiting Liu, Rongfei Yang, Nana Zhang, Qingshan Liu

**Affiliations:** Key Laboratory of Ethnomedicine of Ministry of Education, Center on Translational Neuroscience, School of Pharmacy, Minzu University of China, Beijing, China

**Keywords:** Depression, complementary and alternative medicine, clinical trial, systematic review, umbrella review

## Abstract

**Objective:**

Depression is a major public health problem. Many complementary and alternative medicine (CAM) interventions have been suggested as potential treatments. This umbrella review aimed to assess the efficacy and safety of CAM interventions for treating depression.

**Methods:**

We comprehensively searched for relevant systematic reviews and meta-analyses in the MEDLINE (via PubMed), EMBASE, Web of Science, and Cochrane Library databases. We assessed effectiveness (based on efficacy and changes in depression assessment scale scores) and safety (based on adverse events).

**Results:**

A total of 22 eligible articles were included. Yueju antidepressant and electro-acupuncture (EA) improved depression symptoms better than conventional antidepressants. In addition, omega-3 fatty acids (n-3PUFAs), exercise, manual acupuncture (MA), *Hypericum* mono-preparations, relaxation, and vitamin D showed superior efficacy to placebo and controls. Guipi decotion (GPD) as adjunctive therapy has higher efficacy than conventional antidepressants, and MA and Yueju have a better safety profile than conventional antidepressants.

**Conclusion:**

10 CAMs can effectively improve the condition of patients with clinical depression.

## Introduction

Major depression disorder (MDD) is a significant public health concern that affects more than 300 million individuals worldwide.^1^ It is characterized by depressed mood, reduced interest, impaired cognitive function, loss of appetite, and insomnia, and is associated with high morbidity, mortality, and recurrence rates.^2^,^3^ Globally, retrospective estimates from the World Health Organization’s (WHO) World Mental Health (WMH) Survey suggest that the lifetime prevalence of MDD is approximately 10%. However, a prospective study demonstrated that the lifetime prevalence of MDD was more than twofold higher than that derived from retrospective studies.^4^ In addition, depression increases the risk of development of cardiovascular disease, type 2 diabetes,^5^ and obesity.^6^

Medication is essential for moderate or severe depression.^7^ Antidepressants and psychotherapy are the first line of treatment for patients with MDD. The former include tricyclic antidepressants (TCAs), selective serotonin reuptake inhibitors (SSRIs), and serotonin-norepinephrine reuptake inhibitors (SNRIs),^8^ while common depression-focused psychotherapies include acceptance and commitment therapy, cognitive-behavioral therapy, interpersonal therapy, and psychodynamic and attachment-based therapy.^9^ However, antidepressants have drawbacks such as slow onset, adverse reactions, low efficacy, and patient compliance issues. More than 30% of patients with MDD do not respond to multiple antidepressant trials,^10^ and antidepressants can take up to 6 weeks to become effective.^11^ TCAs produce adverse effects such as constipation, dizziness, and dry eye.^12^ SSRIs and SNRIs are more tolerable but still cause adverse reactions, including difficulty falling asleep, nausea, sexual dysfunction, and diarrhea.^13^,^14^ Despite the proven effectiveness of psychotherapy, its use is restricted by limited access to skilled providers, high cost, fixed hours, its time-consuming nature, and low patient engagement and motivation.^15^,^16^ Therefore, it is vital to find more effective and safer treatments for depression.

Complementary and alternative medicine (CAM) refers to non-mainstream approaches that are not part of traditional Western healthcare or originated outside of standard Western practice.^17^ Several systematic reviews and meta-analyses have shown that CAM can be more effective for depression than placebo or antidepressants.^18^-^21^ In addition, CAM is better tolerated, has lower costs, and has fewer adverse events than conventional antidepressants.^22^ Within this context, we conducted an umbrella review of systematic reviews and meta-analyses to examine the safety and efficacy of CAM for depression, hoping to provide more options for treatment of MDD.

## Methods

This study followed the Preferred Reporting Items for Systematic Reviews and Meta-analysis (PRISMA) standard guidelines.^23^ The protocol for this review was registered prospectively on the International Platform of Registered Systematic Review and Meta-analysis Protocols (INPLASY^®^; protocol number INPLASY202420003).

### Search strategy

We conducted a systematic search of published, peer-reviewed, English-language literature indexed in the MEDLINE (via PubMed), EMBASE, Web of Science, and Cochrane Library databases up to December 2023. The search terms were as follows: (major depression disorder or depression or anxiety) and (systematic review or meta-analysis) and (complementary and alternative therapies) and clinical trials. We included meta-analyses and systematic reviews that assessed the efficacy and safety of treatments in patients with depression. The inclusion criteria were: 1) written in English; 2) systematic review or meta-analysis; 3) included any evaluation of clinical assessment scales for depression or adverse effects and response rates; and 4) published in peer-reviewed journals. Studies were excluded if: 1) they were unpublished; 2) lacked necessary sample data; 3) included patients with a diagnosis of prenatal depression, postpartum depression, post-stroke depression, or other psychiatric disorders; 4) reported insufficient details or outcomes other than the outcomes of interest; or 5) were deemed to have low or critically low evidence quality (see below). We also searched for related articles using keywords and filtering titles.

Two investigators (YL and RY) screened the literature independently. The articles were downloaded, and their abstracts were screened for the inclusion criteria, with irrelevant or duplicate articles deleted. Disagreements were resolved by consensus with the third investigator (NZ). Next, the reference lists of the chosen studies were manually searched for any other relevant studies not found in the initial search. Finally, the full text of the articles deemed eligible was reviewed for data extraction and analysis.

### Data extraction

Three investigators (YL, RY, and NZ) independently selected studies that met the inclusion criteria. The main characteristics of the selected reviews were extracted into a table, including publication year, number of included studies, number of patients, and treatment regimens. Records reporting at least one of the following outcomes were included: 1) efficacy rate/response rate of CAM; 2) depression assessment scales, including the Hamilton Depression Scale (HAMD), Self-rating Depression Scale (SDS), Beck Depression Rating Scale (BDI), etc.; and 3) incidence of adverse events. We also extracted the number of studies, the number of patients, mean difference (MD), relative risk (RR), odds ratio (OR), 95%CI, and heterogeneity (I^2^) from meta-analyses.

### Statistical analysis

The response of patients with depression to CAM was evaluated according to the efficacy rate. CAM-mediated improvement in depressive symptoms was evaluated according to the depression rating scale score, while CAM safety was evaluated based on the incidence of adverse events. For between-study heterogeneity, a percentage of 0-25% was classified as slight, 26-50% as moderate, and 51-75% as substantial. If *I* ^2^ > 50%, a random-effects model was used for the analysis; otherwise, the data were analyzed in a fixed-effects model.^24^

### Quality assessment

We selected the AMSTAR 2 tool to evaluate the quality of articles in systematic reviews and meta-analyses.^25^ The AMSTAR 2 quality appraisal tool consists of 16 items, of which seven are deemed critical, including: 1) protocol registered before commencement of the review (item 2); 2) adequacy of the literature search (item 4); 3) justification for excluding individual studies (item 7); 4) risk of bias from individual studies being included in the review (item 9); 5) appropriation of meta-analytical methods (item 11); 6) consideration of risk of bias when interpreting the results of the review (item 13); and 7) assessment of presence and likely impact of publication bias (item 15). Based on application of the tool, study quality was rated as high (no weaknesses at all or one non-critical weakness), moderate (more than one non-critical weakness), low (one critical flaw with or without non-critical weaknesses), or critically low (more than one critical flaw with or without non-critical weaknesses).

For all analyses, a p-value < 0.05 was considered to indicate a statistically significant difference.

## Results

### Literature search and study selection

**Figure 1:**
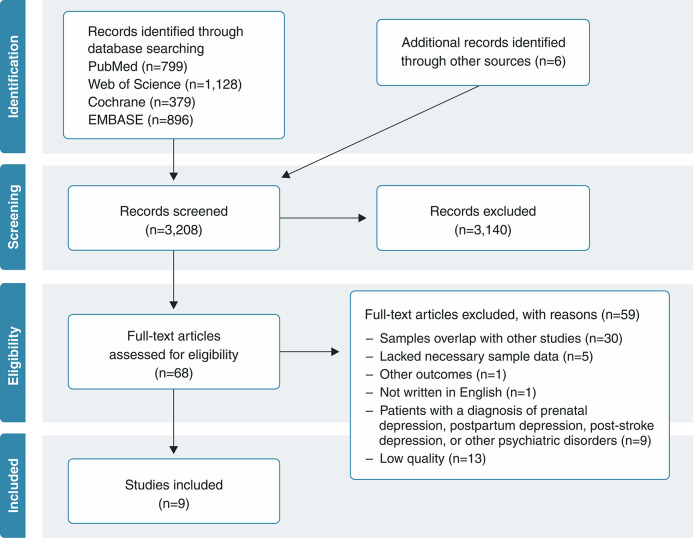
Literature review flowchart.

Overall, a total of 3,208 records were retrieved: 3,202 from databases and six from reference lists of relevant literature. After screening of titles and abstracts, 68 studies were considered eligible for full-text review, of which 46 were excluded for the following reasons: sample overlap with other studies (n=30); lacking necessary sample data (n=5); other outcomes (n=1); other mental disorders (n=9); other language (n=1); or low quality (n=13) (Figure 1). Thus, nine studies were ultimately included in the umbrella review.^18^,^20^,^26^-^32^

As shown in Table 1, 212 clinical trials were included, with treatment groups including 12 CAMs or CAM/conventional therapy combinations (with CAMs as adjunctive therapies). All studies were randomized controlled clinical trials. The measured outcomes covered various depression scales, including the HAMD, Geriatric Depression Scale (GDS), Hospital Anxiety and Depression Scale (HADS), SDS, Montgomery Asberg Depression Rating Scale (MADRS), BDI, Patient Health Questionnaire (PHQ-9), and Clinical Global Impression Index (CGI). Among the included articles, six were considered of high quality and three were considered of moderate quality.

### Depression assessment scales

To assess the effect of CAM on depressive symptoms, we used the reported depression assessment scale findings. A systematic review and meta-analysis of various CAMs for treating depression was conducted, the results of which are summarized in Supplementary Table S1. Electro-acupuncture (EA) (MD: -1.26, 95%CI -2.10 to -0.43, p = 0.003), Yueju antidepressant (MD: -0.92, 95%CI -1.36 to -0.48, p < 0.0001), manual acupuncture (MA) plus SSRI (MD: -1.32, 95%CI -2.09 to -0.55, p = 0.00075), EA plus SSRI (MD: -0.84, 95%CI -1.16 to -0.51, p < 0.00001), Guipi decoction (GPD) plus antidepressants (MD: -3.09, 95%CI -4.11 to -2.07, p < 0.00001), and music therapy plus treatment as usual (MD: -0.66, 95%CI -1.06 to -0.26, p = 0.0069) yielded better outcomes than antidepressants alone or treatment as usual. Seven studies including omega-3 fatty acids (n-3PUFAs) (MD: -0.4, 95%CI -0.64 to -0.16, p = 0.001), exercise (MD: -0.62, 95%CI -0.81 to -0.42, p < 0.0001), MA (MD: -0.56, 95%CI -0.98 to 0.15, p = 0.007), EA (MD: -1.26, 95%CI -2.10 to -0.43, p = 0.003), *Hypericum* mono-preparations (MD: -2.48, 95%CI -3.06 to -1.89, p < 0.0001), relaxation (MD: -0.59, 95%CI -0.94 to -0.24, p = 0.00098), and vitamin D (MD: -0.60, 95%CI -1.19 to -0.01, p = 0.046) reported better outcomes than placebo, no/minimal treatment, and control. Some CAMs were effective for depression, but not as effective as antidepressants; these included MA (MD: -0.23, 95%CI -0.50 to 0.04, p = 0.09) and *Hypericum* mono-preparations (MD: -0.06, 95%CI -0.64 to 0.51, p = 0.8).

### Efficacy rate

Three studies reported the efficacy rate (effectiveness, response, or remission). Detailed characteristics of included studies are listed in Supplementary Table S2. The efficacy rate of GPD plus antidepressants (OR: 4.75, 95%CI 2.66 to 8.51, p < 0.00001) was significantly higher than that of antidepressants, and *Hypericum* mono-preparations (RR: 1.15, 95%CI 1.02 to 1.29, p = 0.02) were significantly better than placebo. In contrast, *Hypericum* mono-preparations (RR: 1.01, 95%CI 0.93 to 1.10, p = 0.9) and n-3PUFAs (OR: 1.39, 95%CI 0.95 to 2.04, p = 0.38) did not differ significantly from antidepressants.

### Adverse effects

We only assessed the adverse effects of CAMs as monotherapy. The results showed that n-3PUFAs (OR: 1.27, 95%CI 0.99 to 1.64, p = 0.06) and *Hypericum* mono-preparations (OR: 0.61, 95%CI 0.28 to 1.31, p = 0.2) were not significantly associated with adverse events. Adverse event scores were lower after MA (MD: -4.32, 95%CI -7.41 to -1.23, p = 0.0061) and the incidence of adverse events was lower with use of Yueju antidepressant (RR: 0.15, 95%CI 0.08 to 0.27, p < 0.00001) than with drug therapy alone (Supplementary Table S3).

## Discussion

This umbrella review appraised the relative effectiveness and safety of several CAM modalities for treatment of major depressive disorder. We attempted to summarize data from published systematic reviews and meta-analyses to determine whether there are CAMs which can be of significant benefit to patients with depression, whether as monotherapy or as adjuncts to conventional antidepressants.

We found that Yueju antidepressant alone and MA as an adjunct to SSRIs had superior efficacy and safety to conventional antidepressants. In addition, *Hypericum* mono-preparations, exercise, relaxation, vitamin D, EA, n-3PUFAs, music therapy plus antidepressants, and GPD plus antidepressants also improved depression symptoms significantly. These results should be interpreted with caution to determine the best therapeutic and adjunctive treatment strategies for depressed patients.

Traditional Chinese Medicine (TCM) is a catchall term for all Chinese ethnic medicines and is the main form of CAM, including herbal medicines (mono-preparations and formulas), acupuncture, and other nonpharmacological therapies, which are multi-component, multi-targeted, and multi-pathway. Herbal medicines have been shown to produce fewer adverse events than conventional antidepressants, and combining herbal medicines with Western medicines shows superior efficacy and safety in treating depression than Western medicines alone.^33^,^34^

The medicinal plant *Hypericum perforatum* (St John’s wort), as assessed in a Cochrane review by Linde et al., shares the same mechanism of action of classical pharmacologic antidepressants.^35^ The major antidepressant component of GPD is ferulic acid.^29^ In one trial included in this review, combining GPD and escitalopram oxalate tablets improved the efficacy of the latter, reduced adverse effects, and improved patients’ sleep quality.^36^ Network pharmacology and molecular docking studies have shown that GPD may act on MDD by modulating neurophysiological processes, vascular morphogenesis, the cAMP responsive element modulator (CREM) pathway, and androgen receptor (AR) signaling crosstalk.^37^ The Yueju recipe has a rapid antidepressant effect similar to that of ketamine, and produces this effect in a protein kinase A (PKA)-cAMP response element-binding protein (CREB) signaling-dependent manner as well as by inducing an increase in adenylate cyclase-activating polypeptide 1 (adenylate cyclase-activating polypeptide 1 [ADCYAP1]/pituitary adenylate cyclase-activating polypeptide [PACAP]).^38^,^39^

The standardization of dosage forms for herbal products derived from TCM (such as GPD) is a critical factor in ensuring the reliability and consistency of clinical trial results. It has been noted that variability in the herbal formulas used across different clinical trials can introduce heterogeneity into the data, complicating the systematic review process.^29^ Regarding Yueju, it is promising to observe that it exhibits rapid antidepressant action comparable to that of ketamine, but, unlike the latter, without producing anxiety-like behavior. The finding that only a specific dose of the ethanol extract of Yueju (1,400 mg/kg in the ICR strain of mice) produces a long-lasting antidepressant effect and reduces immobility time in the tail-hanging and forced swimming tests is significant; no such effect was observed at low (700 mg/kg) or high (2,800 mg/kg) doses. This suggests that dosing is a crucial aspect of the efficacy of herbal treatments, and underscores the need for precise dosing regimens in clinical practice.^38^ The reported side effects of mild headache, dizziness, and thirst associated with Yueju are relatively mild compared to those of most conventional antidepressants, which is an advantage for those seeking alternative treatments.^30^ *Hypericum* preparations, however, exhibit drug-drug interactions with a wide range of conventional medications, which is a considerable concern. These interactions can limit the use of *Hypericum* mono-preparations in patients who are on multiple medications, potentially leading to treatment restrictions and compromising efficacy.^40^

Acupuncture can be subcategorized as MA, EA, or laser acupuncture (LA). MA involves manual manipulation of metal needles to stimulate acupuncture points for therapeutic effect, while EA involves applying a pulsed current through acupuncture needles and LA involves stimulating acupuncture points using low-level laser beams instead of needles. The systematic review included herein suggests that acupuncture could be an effective adjunct to conventional antidepressant therapy.^41^ One study found that adjunctive acupuncture could shorten the latency of response to SSRI treatment, potentially via dopaminergic mechanisms.^42^ EA has been shown modulate the hyperactivity of the hypothalamic-pituitary-adrenal axis, reduce plasma corticosterone levels, decrease central and peripheral tumor necrosis factor (TNF) levels, increase synaptic plasticity and AMPA receptor gene and protein expression, and modulate the expression of fibroblast growth factor in the hippocampus to exert an antidepressant effect.^43^ However, variations in treatment duration and cultural perceptions of acupuncture have significant implications for the outcomes of acupuncture treatment for depression. Chinese studies often follow traditional protocols that may involve longer treatment courses, reflecting the philosophy that acupuncture is a cumulative therapy that requires repeated sessions to achieve optimal results. In contrast, Western studies often use shorter treatment durations due to differences in healthcare practices, expectations and, possibly, the structure of clinical trials. These discrepancies can affect the perceived efficacy of acupuncture, as longer treatment periods might be necessary to observe significant antidepressant effects.^41^ The cultural context in which acupuncture is practiced can influence patient expectations, adherence to treatment, and the perceived effectiveness of therapy. In China, acupuncture is a deeply ingrained aspect of healthcare, and patients may have different expectations and beliefs about its efficacy compared to Western patients. These cultural perceptions can influence the placebo effect and the overall outcomes of treatment. The expertise and training of the acupuncturist are also critical factors in effectiveness and safety. Patients treated by trained and experienced acupuncturists tolerate well and have a lower incidence of adverse events.^42^,^43^

Most of the herbs used in phytopharmacology for depression are available as over-the-counter preparations or dietary supplements. Non-herbal commercially available preparations include various nutraceuticals, such as vitamins (vitamin D and vitamin B), S-adenosylmethionine, amino acids (tyrosine, tryptophan), and trace minerals (zinc, magnesium).^35^ Omega-3 fatty acids have anti-inflammatory properties and are capable of interacting with serotonergic and dopaminergic transmission, which can positively influence depressive states.^44^ An association between vitamin D deficiency and depression has been reported.^45^,^46^ Vitamin D supplementation and increasing serum vitamin D levels can reduce the development of depression, decrease the risk of suicide, and have a beneficial effect on both the onset and prognosis of depression.^47^-^50^ However, the beneficial effects of vitamin D are weak against MDD.^31^ Our results indicate that n-3PUFAs and vitamin D do have an effect on depressive symptoms when compared to placebo, although heterogeneity is high and the results are less precisely estimated. Given that n-3PUFAs and vitamin D are also present in the diet, it is essential that future high-quality studies control for subjects’ dietary intake.^26^,^31^

Exercise is most helpful for refractory depression, unipolar depression, and posttraumatic stress disorder (PTSD).^51^ Relaxation and psychotherapy can be used together in stepped care, with relaxation as the first-line treatment and psychotherapy as the second-line treatment.^18^ However, exercise is better suited to those who are motivated and physically fit and have mild to moderate depression. Exercise-based programs can be specifically tailored for individuals with depression.^52^ There are no standardized dosage guidelines for the progressive muscle relaxation used in relaxation therapy, as various courses and delivery methods are available, and the severity of depression for which relaxation therapy is appropriate is not well-defined.^18^ Regarding music therapy, there is currently a shortage of trained specialists worldwide, and the number of practitioners varies significantly from country to country.^32^

In conclusion, our findings suggest that a variety of complementary and alternative therapies can improve or assist in improving depressive symptoms. However, these are not the only solutions for depression, and these treatments may not be suitable for all individuals with depression. This issue also applies to conventional treatments, as pharmacotherapy and psychotherapies may not be appropriate for everyone. Therefore, determining the applicability of CAMs in depression should be a focus of subsequent research efforts.

This study had some limitations. First, most of the meta-analyses on CAM were either very small in number or lacked relevant meta-analyses, resulting in coverage of only a limited number of CAM modalities and entry of low-quality articles. Second, due to the limited number of relevant RCTs, most of the studies were mixed with studies on post-stroke depression, prenatal depression, postnatal depression, or simply depressed-mood samples, making their inclusion impossible. Finally, only a few of the included meta-analyses examined the safety of CAM, resulting in limited data.

## Disclosure

The authors report no conflicts of interest.

## Figures and Tables

**Table 1:** Characteristics of included studies

Study	Conditions	Studies included (n)	Study duration (weeks)	Participants (n)	Outcomes	Study quality
Appleton et al.^26^	n-3PUFAs	35	4-16	1,944	BDI, GDS, HADS, MADRS, PHQ-9, efficacy rate, adverse effects	High
Cooney et al.^27^	Exercise	39	6-34	2,326	HAMD	High
Linde et al.^28^	*Hypericum* mono-preparations	26	6-18	3,320	HAMD, CGI, DS, efficacy rate, adverse effects	High
Smith et al.^20^	Acupuncture	64	1-12	7,104	HAMD, MADRS, PHQ-9, CGI-S, BDI, adverse effects	High
Sheng et al.^29^	A combination of GPD plus antidepressants	9	4-12	650	HAMD, efficacy rate	Moderate
Yu et al.^30^	Yueju antidepressant	12	1-8	770	HAMD, SCL-90, SDS, adverse effects	Moderate
Jorm et al.^18^	Relaxation	11	4-24	-	BDI, CES-D, GDS, HADS, RADS, CDI	High
Shaffer et al.^31^	Vitamin D	7	-	3,191	BDI, HRSD	Moderate
Aalbers et al.^32^	Music therapy	9	6-12	411	HAMD, BDI	High

BDI = Beck Depression Inventory; CDI = adapted Children’s Depression Inventory; CES-D = Center for Epidemiologic Studies Depression Scale; CGI = Clinical Global Impression Index; DS = Depression Scale von Zerssen; GDS = Geriatric Depression Scale; GPD = Guipi decoction; HADS = Hospital Anxiety and Depression Scale; HAMD = Hamilton Depression Scale; HRSD = Hamilton Rating Scale of Depression; MADRS = Montgomery-Åsberg Depression Rating Scale; n-3PUFAs = omega-3 fatty acids; PHQ-9 = Patient Health Questionnaire; RADS = Reynolds Adolescent Depression Scale; SCL-90 = Self-reporting Inventory; SDS = Self-Rating Depression Scale.

## Data Availability

The original contributions presented in the study are included in the article material. Further inquiries can be directed to the corresponding authors.
