# Supplementary material for: The efficacy and safety of complementary and alternative medicine for depression: an umbrella review
Source: Braz J Psychiatry. 2024 Nov 25;46:e20243705. doi: 10.47626/1516-4446-2024-3705 (PMC11773322; doi:10.47626/1516-4446-2024-3705)
Supplement: Supplementary file 1 [file bjp-46-e20243705-suppl1.pdf]

http://doi.org/10.47626/1516-4446-2024-3705

Supplementary Table S1 Results of pairwise meta-analyses of CAMs for depression

Study	Comparative therapies	Reference therapies	Number of studies	Number of patients	MD	95% CI	I^2	P-value
Appleton et al.[1]	n-3PUFAs	Placebo	27	1,848	-0.4	-0.64 to -0.16	81%	0.001
Cooney et al.[2]	Exercise	Control (no treatment or placebo)	35	1,353	-0.62	-0.81 to -0.42	63%	< 0.0001
		Pharmacological treatments	4	300	-0.11	-0.34, 0.12	0%	0.34
Smith et al.[3]	MA	No treatment	5	458	-0.56	-0.98 to 0.15	62%	0.007
		SSRI	16	1,570	-0.23	-0.50 to 0.04	84%	0.09
	EA	No treatment	1	30	-1.26	-2.10 to -0.43	-	0.003
		SSRI	5	197	-0.47	-0.85 to -0.10	34%	0.013
Linde et al.[4]	*Hypericum* mono-preparations	Placebo	12	1,824	-2.48	-3.06 to -1.89	81%	< 0.0001
		Antidepressant	12	1,729	-0.06	-0.64 to 0.51	45.5%	0.8
Yu et al.[5]	Yueju antidepressant	Antidepressants	11	1,722	-0.92	-1.36 to -0.48	7%	< 0.0001
Jorm et al.[6]	Relaxation	Control (no/minimal treatment)	5	136	-0.59	-0.94 to -0.24	49%	0.00098
Shaffer et al.[7]	Vitamin D	Control (no injection/placebo)	2	149	-0.60	-1.19 to -0.01	-	0.046
Smith et al.[3]	MA plus SSRI	SSRI	8	539	-1.32	-2.09 to -0.55	93%	0.00075
	EA plus SSRI	SSRI	5	274	-0.84	-1.16 to -0.51	33%	< 0.00001

Sheng et al.[8]	A combination of GPD plus antidepressants	Antidepressants	8	560	-3.09	-4.11 to -2.07	71%	< 0.00001
Aalbers et al.[9]	Music therapy plus treatment as usual	Treatment as usual	4	219	-0.98	-1.69 to -0.27	83%	0.0069

EA = electro-acupuncture; GPD = Guipi Decoction; MA = manual acupuncture; n-3PUFAs = omega-3 fatty acids.

Supplementary Table S2 Results of paired meta-analyses of effective rate/response rate after CAM treatment in depression

Study	Comparative therapies	Reference therapies	Number of studies	Number of patients	RR/OR	95% CI	I^2	P-value
Appleton et al.[1]	n-3PUFAs	Placebo	15	611	1.39	0.95-2.04	6%	0.38
Linde et al.[4]	*Hypericum* mono-preparations	Antidepressants	13	2,044	1.01	0.93-1.10	3.0%	0.9
		Placebo	6	1,241	1.15	1.02-1.29	48.0%	0.02
Sheng et al.[8]	A combination of GPD plus antidepressants	Antidepressants	7	480	4.75	2.66-8.51	0.0%	< 0.00001

GPD = Guipi Decoction; n-3PUFAs = omega-3 fatty acids; OR = odds ratio; RR = relative risk.

Supplementary Table S3 Results of pairwise meta-analyses for adverse effects

Study	Comparative therapies	Reference therapies	Number of studies	Number of patients	MD	95% CI	I^2	P-value
Smith et al.[3]	MA	SSRI	3	481	-4.32	-7.41 to -1.23	97%	0.0061

Study	Comparative therapies	Reference therapies	Number of studies	Number of patients	RR/OR	95% CI	I^2	P-value
Appleton et al.[1]	n-3PUFAs	Placebo	19	1,503	1.27	0.99-1.64	2%	0.06
Linde et al.[4]	*Hypericum* mono-preparations	Placebo	24	2,626	0.61	0.28-1.31	0%	0.2
Yu et al.[10]	Yueju antidepressant	Antidepressants	9	603	0.15	0.08-0.27	23%	< 0.00001

MA = manual acupuncture; n-3PUFAs = omega-3 fatty acids; RR = relative risk; SSRI = selective serotonin reuptake inhibitors.

Supplementary Table S4 Result of AMSTAR 2 appraisal

Study	Conditions	Item 2	Item 4	Item 7	Item 9	Item 11	Item 13	Item 15	Number of other noncritical entries failed	Result
Zhang et al.[11]	Shuganjieyu capsule	✓	✓	✓	✓	✓	✓	✕	> 1	Low
Yang et al.[12]	Wendan Decoction	✓	✕	✕	✓	✕	✓	✕	-	Critically low
Meekums et al.[13]	Dance movement therapy	✓	✓	✓	✓	✓	✓	✕	-	Low
Dib et al.[14]	Probiotics	✓	✕	✓	✓	✕	✓	✕	-	Critically low
Galizia et al.[15]	*S*-adenosyl methionine	✓	✓	✓	✓	✓	✓	✕	-	Low
Dai et al.[16]	Saffron (*Crocus sativus* L.)	✓	✓	✓	✓	✓	✓	✕	-	Low
Sun et al.[17]	Chai Hu Shu Gan San	✓	✓	✓	✓	✕	✕	✕	-	Critically low

Barbato et al.[18]	Couples therapy	✓	✓	✓	✓	✓	✓	✕	-	Low
Miao et al.[19]	Mindfulness yoga	✓	✓	✓	✓	✓	✓	✕	-	Low
Rotella et al.[20]	Homeopathic remedies	✓	✕	✓	✓	✓	✓	✓	-	Low
Appleton et al.[1]	Omega-3 fatty acids	✓	✓	✓	✓	✓	✓	✓	0	High
Cooney et al.[2]	Exercise	✓	✓	✓	✓	✓	✓	✓	1	High
Linde et al.[4]	*Hypericum* mono-preparations	✓	✓	✓	✓	✓	✓	✓	1	High
Smith et al.[3]	Acupuncture	✓	✓	✓	✓	✓	✓	✓	1	High
Sheng et al.[8]	Guipi Decoction plus antidepressants	✓	✓	✓	✓	✓	✓	✓	> 1	Moderate
Yu et al.[5]	Yueju antidepressant	✓	✓	✓	✓	✓	✓	✓	> 1	Moderate
Ng et al.[21]	Curcumin	✓	✓	✓	✓	✓	✕	✕	-	Critically low
Schefft et al.[22]	Add-on nutritional supplements	✓	✕	✓	✓	✓	✓	✓	-	Low
Jorm et al.[6]	Relaxation	✓	✓	✓	✓	✓	✓	✓	1	High
Shaffer et al.[7]	Vitamin D	✓	✓	✓	✓	✓	✓	✓	> 1	Moderate

Mukai et al.[23]	Inositol	✓	✓	✓	✕	✓	✓	✓	-	Low
Aalbers et al.[9]	Music therapy	✓	✓	✓	✓	✓	✓	✓	0	High

The AMSTAR-2 quality assessment tool has 16 items, of which 7 are critical items, including item 2, item 4, item 7, item 9, item 11, item 13, and item 15.

Rating overall confidence in the results of the review

• High

No or one non-critical weakness: the systematic review provides an accurate and comprehensive summary of the results of the available studies that address the question of interest.

• Moderate

More than one non-critical weakness: the systematic review has more than one weakness but no critical flaws. It may provide an accurate summary of the results of the available studies that were included in the review.

• Low

One critical flaw with or without non-critical weaknesses: the review has a critical flaw and may not provide an accurate and comprehensive summary of the available studies that address the question of interest.

• Critically low

More than one critical flaw with or without non-critical weaknesses: the review has more than one critical flaw and should not be relied on to provide an accurate and comprehensive summary of the available studies

References

1. Appleton KM, Voyias PD, Sallis HM, Dawson S, Ness AR, Churchill R, et al. Omega-3 fatty acids for depression in adults. Cochrane Database Syst Rev. 2021;11:CD004692.
2. Cooney GM, Dwan K, Greig CA, Lawlor DA, Rimer J, Waugh FR, et al. Exercise for depression. Cochrane Database Syst Rev. 2013;2013:CD004366.
3. Smith CA, Armour M, Lee M S, Wang L-Q, Hay PJ. Acupuncture for depression. Cochrane Database Syst Rev. 2018;3:CD004046.
4. Linde K, Berner MM, Kriston L. St John's wort for major depression. Cochrane Database Syst Rev. 2008;2008:CD000448.
5. Yu YH, Song HY, Liu JG, Wang PL, Wang CL. Efficiency and safety of yueju antidepressant for primary depression patients: a meta-analysis of randomized controlled trials. J Herb Med. 2021;28:100329.
6. Jorm AF, Morgan AJ, Hetrick SE. Relaxation for depression. Cochrane Database Syst Rev. 2008:CD007142.
7. Shaffer JA, Edmondson D, Wasson LT, Falzon L, Homma K, Ezeokoli N, et al. Vitamin D supplementation for depressive symptoms: a systematic review and meta-analysis of randomized controlled trials. Psychosom Med. 2014;76:190-6.
8. Sheng CX, Chen ZQ, Cui HJ, Yang AL, Wang C, Wang Z, et al. Is the Chinese medicinal formula Guipi Decoction () effective as an adjunctive treatment for depression? A meta-analysis of randomized controlled trials. Chin J Integr Med. 2017;23:386-95.
9. Aalbers S, Fusar-Poli L, Freeman RE, Spreen M, Ket JC, Vink AC, et al. Music therapy for depression. Cochrane Database Syst Rev. 2017;11:CD004517.
10. Yu J, Xu F-Q. Clinical efficacy and safety of Guipi decoction combined with escitalopram oxalate tablets in patients with depression. World J Clin Cases. 2023;11:7017-25.
11. Zhang X, Kang D, Zhang L, Peng L. Shuganjieyu capsule for major depressive disorder (MDD) in adults: a systematic review. Aging Ment Health. 2014;18:941-53.
12. Yang Y, Chen R, Li C, Zheng Q, Lv Y, Li L, et al. A Synthetic external control study comparing the clinical efficacy of wendan decoction and 19 antidepressants. Int J Neuropsychopharmacol. 2023;26:739-46.
13. Meekums B, Karkou V, Nelson EA. Dance movement therapy for depression. Cochrane Database Syst Rev. 2015;2015:CD009895.
14. El Dib R, Periyasamy AG, Barros JL, França CG, Senefonte FL, Vesentini G, et al. Probiotics for the treatment of depression and anxiety: A systematic review and meta-analysis of randomized controlled trials. Clin Nutr ESPEN. 2021;45:75-90.
15. Galizia I, Oldani L, Macritchie K, Amari E, Dougall D, Jones TN, et al. S-adenosyl methionine (SAMe) for depression in adults. Cochrane Database Syst Rev. 2016;10:CD011286.
16. Dai L, Chen L, Wang W. Safety and efficacy of saffron (Crocus sativus L.) for treating mild to moderate depression: a systematic review and meta-analysis. J Nerv Ment Dis. 2020;208:269-76.
17. Sun Y, Xu X, Zhang J, Chen Y. Treatment of depression with Chai Hu Shu Gan San: a systematic review and meta-analysis of 42 randomized controlled trials. BMC Complement Altern Med. 2018;18:66.
18. Barbato A, D'Avanzo B, Parabiaghi A. Couple therapy for depression. Cochrane Database Syst Rev. 2018;6:CD004188.
19. Miao C, Gao Y, Li X, Zhou Y, Chung JW, Smith GD. The effectiveness of mindfulness yoga on patients with major depressive disorder: a systematic review and meta-analysis of randomized controlled trials. BMC Complement Med Ther. 2023;23:313.
20. Rotella F, Cassioli E, Falone A, Ricca V, Mannucci E. Homeopathic remedies in psychiatric disorders: a meta-analysis of randomized controlled trials. J Clin Psychopharmacol. 2020;40:269-75.

21. Ng QX, Koh SSH, Chan HW, Ho CYX. Clinical use of curcumin in depression: a meta-analysis. J Am Med Dir Assoc. 2017;18:503-8.
22. Schefft C, Kilarski LL, Bschor T, Köhler S. Efficacy of adding nutritional supplements in unipolar depression: a systematic review and meta-analysis. Eur Neuropsychopharmacol. 2017;27:1090-109.
23. Mukai T, Kishi T, Matsuda Y, Iwata N. A meta-analysis of inositol for depression and anxiety disorders. Hum Psychopharmacol. 2014;29:55-63.
